# Loss-of-function variant in *SPIN4* causes an X-linked overgrowth syndrome

**DOI:** 10.1172/jci.insight.167074

**Published:** 2023-05-08

**Authors:** Julian C. Lui, Jacob Wagner, Elaine Zhou, Lijin Dong, Kevin M. Barnes, Youn Hee Jee, Jeffrey Baron

**Affiliations:** 1 Section on Growth and Development, Eunice Kennedy Shriver National Institute of Child Health and Human Development, and; 2 Genetic Engineering Core, National Eye Institute, National Institute of Health, Bethesda, Maryland, USA.

**Keywords:** Development, Genetics, Bone development, Epigenetics, Genetic diseases

## Abstract

Overgrowth syndromes can be caused by pathogenic genetic variants in epigenetic writers, such as DNA and histone methyltransferases. However, no overgrowth disorder has previously been ascribed to variants in a gene that acts primarily as an epigenetic reader. Here, we studied a male individual with generalized overgrowth of prenatal onset. Exome sequencing identified a hemizygous frameshift variant in Spindlin 4 (*SPIN4*), with X-linked inheritance. We found evidence that *SPIN4* binds specific histone modifications, promotes canonical WNT signaling, and inhibits cell proliferation in vitro and that the identified frameshift variant had lost all of these functions. Ablation of *Spin4* in mice recapitulated the human phenotype with generalized overgrowth, including increased longitudinal bone growth. Growth plate analysis revealed increased cell proliferation in the proliferative zone and an increased number of progenitor chondrocytes in the resting zone. We also found evidence of decreased canonical Wnt signaling in growth plate chondrocytes, providing a potential explanation for the increased number of resting zone chondrocytes. Taken together, our findings provide strong evidence that S*PIN4* is an epigenetic reader that negatively regulates mammalian body growth and that loss of *SPIN4* causes an overgrowth syndrome in humans, expanding our knowledge of the epigenetic regulation of human growth.

## Graphical abstract

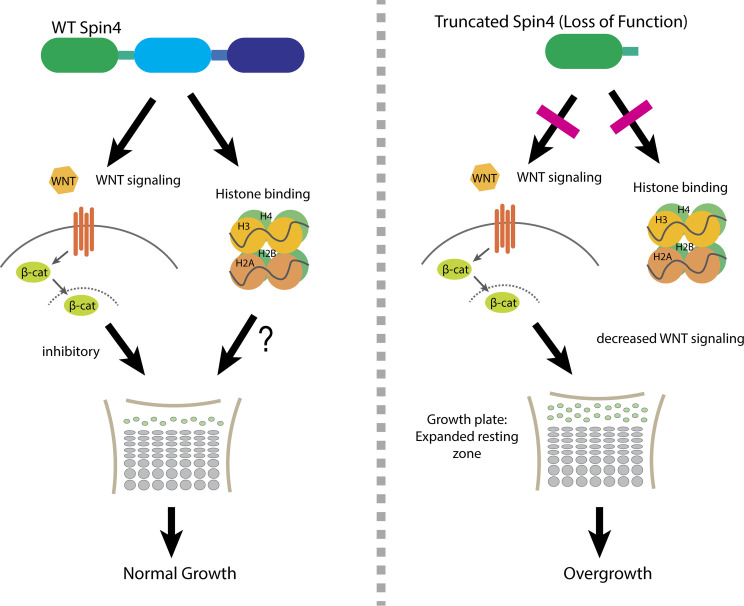

## Introduction

Generalized overgrowth syndromes are rare genetic disorders characterized by excessive body growth during fetal life and/or childhood, often presenting with tall stature. These disorders typically arise from germline variants that promote cell proliferation in multiple tissues. The clinical presentation of each disorder depends, in part, on which specific tissues are affected (1). If proliferation is increased in the skeletal growth plate, the clinical presentation will include increased bone length and, therefore, tall stature, whereas increased growth of the brain results in macrocephaly and increased growth of visceral organs produces organomegaly.

Overgrowth disorders can arise from abnormalities in a variety of genes that regulate proliferation. For example, Beckwith-Wiedemann syndrome can result from increased expression of insulin-like growth factor 2 (*IGF2*) (2), a paracrine growth factor, and/or from decreased expression or inactivating variants in cyclin-dependent kinase inhibitor 1c (*CDKN1C*) (3), which regulates cell cycle progression. Interestingly, multiple different overgrowth syndromes are caused by variants in genes affecting epigenetic pathways (4). For example, Weaver syndrome and Sotos syndrome are caused by pathogenic variants in histone methyltransferases *EZH2* (5) and *NSD1* (6), respectively, while Tatton-Brown-Rahman syndrome is caused by pathogenic variants in a DNA methyltransferase, *DNMT3A* (7). All 3 of these syndromes involve genes that act primarily as epigenetic writers, which add modifications to chromatin. To date, no overgrowth syndrome has been ascribed to a gene that acts primarily as epigenetic erasers, which remove these modifications, or epigenetic readers, which recognize and bind to these modifications to mediate downstream cellular effects.

Spindlin family member 4 (*SPIN4*) is a member of the SPIN/SSTY family. Little is known about the function of *SPIN4*, to date. It shares structural features, including 3 tandem Tudor-like domains, with *SPIN1*, an epigenetic reader that recognizes histone H3 trimethylated at lysine-4 (8, 9) and H3 asymmetrically dimethylated at arginine-8 (10). *SPIN1* has also been shown to stimulate Wnt signaling and promote cell proliferation and cancer progression (11, 12).

We evaluated a male individual with generalized overgrowth of prenatal onset, which, at the time of clinical presentation, included extreme tall stature, enlarged liver and spleen, and macrocephaly but no developmental delay. Exome sequencing identified a protein-truncating frameshift variant in *SPIN4*. In mice, ablation of *Spin4* resulted in overgrowth of multiple organs and overall body size, recapitulating the human phenotype. We found that WT *SPIN4* was able to bind specifically modified histone H3, promote canonical WNT signaling, and inhibit cell proliferation in vitro, but we also found that the variant of *SPIN4*, which lacks 2 of the 3 Tudor-like domains, had lost all of these functions. The findings indicate that *SPIN4* normally serves as an epigenetic reader that negatively regulates body size in mice and humans and, therefore, that loss of function of *Spin4*/*SPIN4* increases somatic growth.

## Results

### Proband and the family.

**Figure 1:**
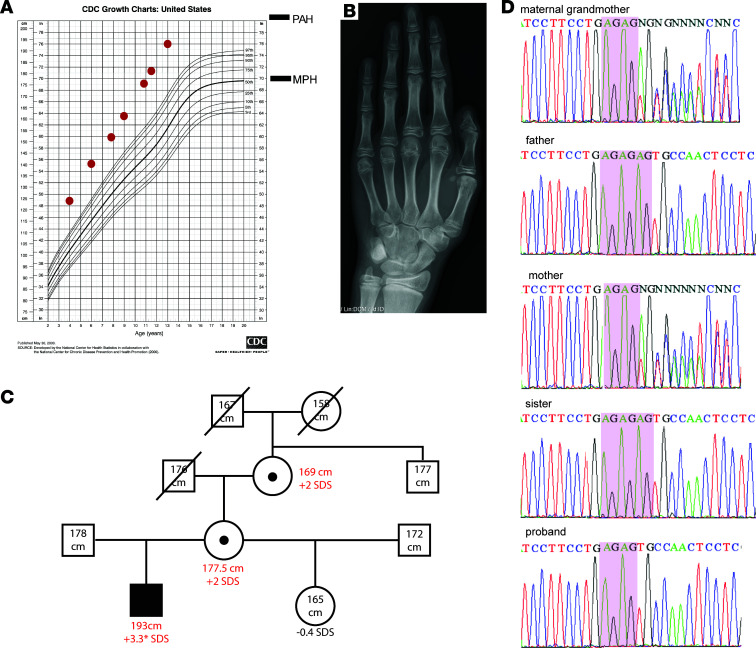
Patient with a new overgrowth syndrome. (**A**) Proband’s growth chart for stature. MPH, midparental height. PAH, predicted adult height. (**B**) Proband’s bone age x-ray. The bone age x-ray was read as 13 years, 6 months, at chronological age 12 years, 5 months. (**C**) Pedigree of the family. Closed circles and rectangles represent affected subjects with height gain. Open circles and rectangles represent unaffected subjects. SDS height gain in red was calculated by subjects’ current height SDS minus subject’s midparental height SDS, except for proband’s height gain (*), which was calculated by proband’s predicted adult height SDS minus subject’s midparental height. (**D**) Sanger sequencing of the frameshift variant (c.312_313AGdel) in the family. MGM, maternal grandmother. AG repeats are marked in pink boxes.

The proband was a 13-year-old boy who was referred for evaluation of an overgrowth syndrome. He was born large for gestational age, with a birth weight of 5.85 kg (+4.3 standard deviation score [SDS]) and a length of 62 cm (+4.8 SDS). The mother had no history of gestational diabetes. He has been growing remarkably with height tracking between +4.5 and +5 SDS, although his midparental height was +1.2 SDS (Figure 1A). His bone age was advanced by 1 year: 13 years, 6 months at a chronological age of 12 years, 5 months (Figure 1B) with a Bayley-Pinneau–predicted adult height of 203 cm. Because of his expected adult tall stature, he underwent epiphysiodesis at 13 years old. His weights were between +2 and +2.8 SDS. He reached Tanner stage 4 (13) pubic hair at the age of 13 years, indicating normal pubertal timing. His head circumferences have been greater than the 97th percentile since birth. He was diagnosed with hydrocephalus without an increase of intracranial pressure on brain ultrasound at 6 months that did not require any intervention. He had normal psychomotor development and intelligence. The proband speaks 3 different languages fluently and is particularly good at math.

Because of his excessive linear growth, he had undergone a comprehensive evaluation. His brain MRI was unremarkable except for a residual structure of Rathke’s pouch. CT of the abdomen showed increased liver and spleen size. His thyroid-stimulating hormone (TSH) and free thyroxine (T4) were normal. His IGF-1 was 179 ng/mL and testosterone was 58 ng/dL, both normal for Tanner stage 2. Repeat IGF-1 levels at different ages were also consistently unremarkable. Serum IGF-2 levels were normal in the proband and other affected family members (Supplemental Figure 1; supplemental material available online with this article; https://doi.org/10.1172/jci.insight.167074DS1). Karyotype, mutation analysis in *NSD1* for Sotos syndrome, and chromosome 11p15 analysis (multiplex ligation–dependent probe amplification [MLPA] and methylation) for Beckwith-Wiedemann syndrome were normal.

On physical exam, he was noted to have protruding ears, mildly wide-spaced nipples, large flat feet, and long arms. He showed down-slanting deep-set eyes and a prominent philtrum. He had no evidence of macroglossia, joint hypermobility, scoliosis, or hemihypertrophy.

Family history revealed that his mother was 177.5 cm (+2.2 SDS) and his father was 178 cm (+0.2 SDS). His mother’s birth weight was 4.5 kg (weight +1.9 SDS) and length was 55 cm (length +2 SDS). The proband’s half-sister had a normal height. Interestingly, both the maternal grandmother and mother had adult heights 2 SDS greater than their midparental heights (Figure 1C). The proband had a predicted adult height +3.3 SDS greater than his midparental height prior to his epiphysiodesis.

### SNP array and exome sequencing.

Because the family pedigree suggested an X-linked semidominant condition, we performed single nucleotide polymorphism (SNP) array and exome sequencing to identify the underlying genetic cause. SNP array showed no significant copy number variants. However, exome sequencing identified a hemizygous frameshift variant (NM_001012968.3, c.312_313AGdel) in *SPIN4* on the X-chromosome (Figure 1D), which was also present in the maternal grandmother and mother but not in other family members. No protein-truncating or loss-of-function variants in *SPIN4* were found in the general population data (gnomAD v.2.1.1, https://gnomad.broadinstitute.org/). The proband had no other strong candidate variants for overgrowth that occurred de novo or were inherited in a recessive mode, such as homozygous or compound heterozygous variants. Furthermore, any autosomal dominant variants passed from maternal grandmother to the mother and proband were (a) benign or (b) not in a gene known to regulate somatic growth. The proband had no significant variants in genes known to cause overgrowth syndrome.

### Loss of function in Spin4 frameshift variant.

**Figure 2:**
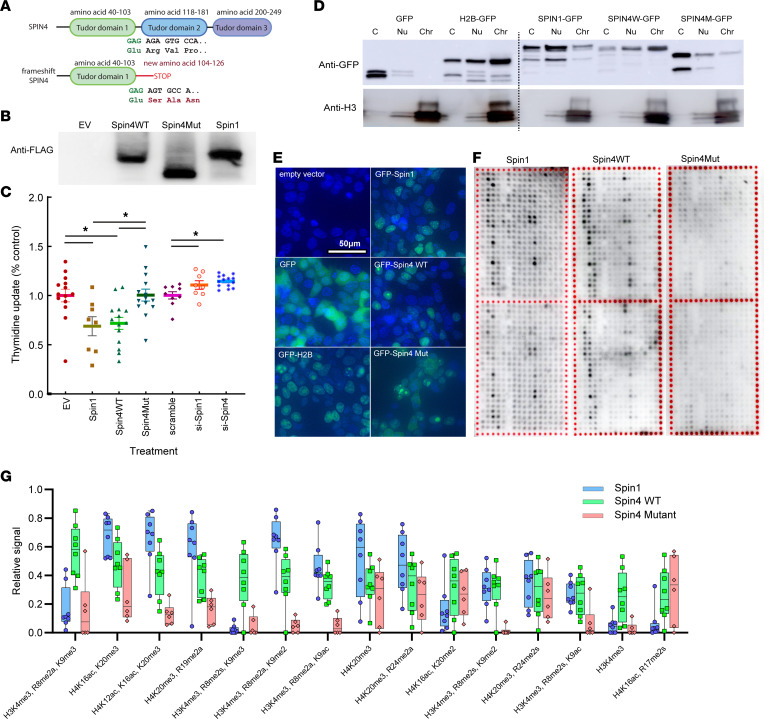
Loss of function in Spin4 frameshift variant. (**A**) Schematic diagram showing the location of the frameshift relative to the 3 Tudor domains. (**B**) Western blot with anti-FLAG antibody confirmed a change in molecular size of the frameshift SPIN4 variant. The experiment was repeated 3 times with similar results. (**C**) Tritiated thymidine uptake in HEK293 cells showed that both SPIN1 and SPIN4 WT suppress proliferation. SPIN4 mutant (Mut) did not show any suppression of proliferation compared with empty vector (EV). Consistently, siRNA against *SPIN1* or *SPIN4* increased proliferation compared with scrambled siRNA. *P < 0.05, 1-way ANOVA, *n* = 8–14. (**D** and **E**) GFP-tagged proteins were transfected in HEK293 cells, and subcellular localization was examined by fractionation followed by anti-GFP Western blot (**D**) or fluorescence microscopy (**E**). H2B-GFP was used as a control for chromatin-bound protein. Anti–histone H3 was used to assess purity of chromatin-bound protein fraction. C, cytoplasmic; Nu, nuclear; chr, chromatin-bound. The experiment was repeated 3 times with similar results. (**F** and **G**) Histone peptide arrays were used to assess histone binding properties of human SPIN1, SPIN4 WT, and Mut. Each spot on the array contains a peptide portion of a histone that has undergone 1 or more specific posttranslational modifications. Darker color indicates greater binding of the indicated Spin protein to that modified peptide. The upper and lower halves of each array are used as technical replicates. Both SPIN1 and SPIN4 WT bind to modified histone peptides, while SPIN4 Mut showed substantially diminished binding. Peptides were ranked based on average binding to SPIN4 WT across 8 arrays and a box-and-whisker plot was generated. The line inside the box represents the median; upper and lower boundary of the boxes represents the 25th and 75th percentile, respectively; whiskers represent the 5th and 95th percentile (*n* = 8).

The identified variant is located shortly after the first of the 3 Tudor domains in the *SPIN4* gene and is predicted to cause a frameshift and protein truncation of the SPIN4 protein with an intact Tudor domain 1, followed by 22 new amino acid residues at the carboxyl-terminus (Figure 2A). Expression of *SPIN4* with the identified variant in HEK293 cells resulted in a protein with reduced molecular weight, confirming the predicted translation of a truncated protein (Figure 2B). We next investigated the effect of SPIN1 and SPIN4 expression on cell proliferation. Compared with the empty vector, overexpression of *SPIN1* or WT *SPIN4*, but not *SPIN4* carrying the truncating variant, suppressed HEK293 cell proliferation (Figure 2C). In contrast, siRNA against *SPIN1* or *SPIN4* showed a modest increase in cell proliferation compared with scrambled siRNA (Figure 2C). These data suggest that *SPIN4* negatively regulates cell proliferation and that the identified frameshift variant abolishes this function.

Previous studies have shown that SPIN1 binds to modified histones (8, 10). A similar property has been reported for SPIN4 but is less well elucidated (14). Therefore, we next performed subcellular fractionation in HEK293 cells to explore the cellular locations of SPIN proteins, and we found that GFP-labeled SPIN1 was primarily located in the nucleus, WT SPIN4 was predominantly found in the chromatin-bound fraction, and the majority of the truncated SPIN4 protein remained in the cytoplasm (Figure 2D). Similarly, fluorescence microscopy showed GFP signal in the cell nucleus for GFP–histone H2B, GFP-SPIN1, and GFP-SPIN4. In contrast, the control GFP signal was diffusely spread out in the cell body, and GFP-truncated SPIN4 was either diffusely spread out in the cell body or clumped in the nucleus (Figure 2E). Finally, we expressed SPIN1, WT, or truncated SPIN4 labeled with an IgG Fc tag and assessed histone binding using a high-throughput histone array (Figure 2, F and G). We found that SPIN1 and WT SPIN4 bound to similarly modified histone peptides, such as H3K4me3 and H4K20me3, while the interaction was significantly diminished with the truncated SPIN4 protein. Taken together, these data indicate that WT SPIN4 binds specifically modified histones and that the frameshift variant identified in the proband produced a truncated protein that dramatically decreased this histone-binding capacity.

### Loss of Spin4 causes overgrowth in mice.

**Figure 3:**
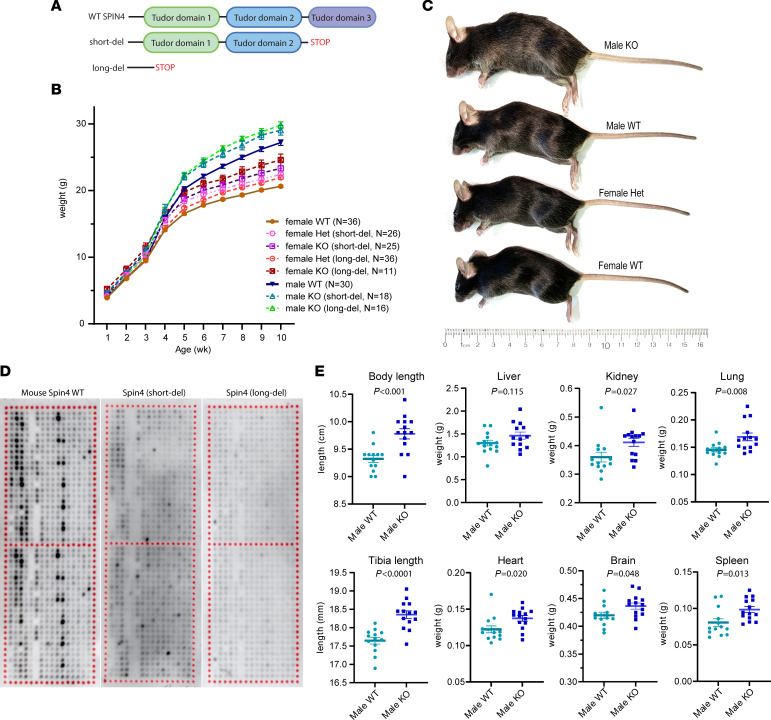
Loss of Spin4 causes overgrowth in mouse. (**A**) Schematic diagram showing the location of Spin4 deletion in 2 lines of mice generated. One line with a short deletion has only Tudor domain 3 removed, while the other line with a long deletion has all 3 Tudor domains removed. (**B**) Growth chart showing male and female WT mice, male hemizygous (KO), and female heterozygous (Het) or homozygous (KO) in both lines. Body weight is significantly increased in both lines of mice in both males and females (2-way ANOVA for age and genotype, *P* < 0.01 for WT versus KO in both sexes, *n* = 11–36). (**C**) Representative image of male KO and female heterozygous mice compared with WT littermates at age 10 weeks. (**D**) Histone array showed that both the long and short deletion of mouse *Spin4* demonstrated loss of histone peptide binding. Each spot on the array contains a peptide that represents a peptide portion of a histone that has undergone 1 or more specific posttranslational modifications. Darker color indicates greater binding of the indicated Spin protein to that modified peptide. The upper and lower halves of each array are used as technical replicates. (**E**) Body and tibia length and organ weights were measured in 10-week-old WT and KO (data combined from long deletion and short deletion) males. The weights of all organs except liver weight were significantly increased in the KO male. Significance was found with Student’s *t* test (*n* = 14–16).

To assess causality between *SPIN4* variant and the overgrowth phenotype of our patient, we generated mouse models with truncating mutations in *Spin4*. We did not introduce the exact 2 nucleotide (AG) deletion that was found in our patient because the difference in codon usage between humans and mice would result in different residues and a different translation-termination site following the frameshift. Instead, we used CRISPR/Cas9 to generate 2 lines of mice, each carrying a different truncating mutation in *Spin4*. One of the 2 lines carries a truncating mutation downstream of the second Tudor domain, while the other line carries a truncating mutation before the start of the first Tudor domain (Figure 3A). Mice from both lines were viable, fertile, and born at the expected Mendelian ratio. Importantly, male mice hemizygous for either the short or long deletion (hereafter referred to as male KO) showed increased body weight (Figure 3, B and C) compared with male WT littermates. Similarly, female mice heterozygous for either deletion also showed increased body weight compared with WT female littermates (Figure 3B). The body weight was significantly greater in homozygous compared with heterozygous females, indicating an effect of gene dosage. Because *Spin4* is not known to escape X-inactivation (15), the growth of heterozygous mice may represent the mixed effect of cells with a transcriptionally active WT *Spin4* allele and those with a transcriptionally active mutant allele.

At 10 weeks of age, an increase in overall body length, tibia length, and most organ weights was observed in male KO mice compared with WT male littermates (Figure 3E). The increases in the mass of these organs was proportional to the increase in overall body mass (Supplemental Figure 2), suggesting that loss of *Spin4* induces uniform overgrowth in multiple tissues. Histone arrays confirmed that both the long and short deletion of mouse *Spin4* greatly diminished or abolished histone binding (Figure 3D). Our findings in the mouse models indicate that *Spin4* is a negative regulator of mammalian body growth and support the hypothesis that hemizygous loss of *Spin4* function contributes to the overgrowth phenotype in our patient and that heterozygous loss of *Spin4* function contributes to the height gain observed in the patient’s mother and maternal grandmother.

### Expanded resting zone of the growth plate in Spin4-KO mice.

**Figure 4:**
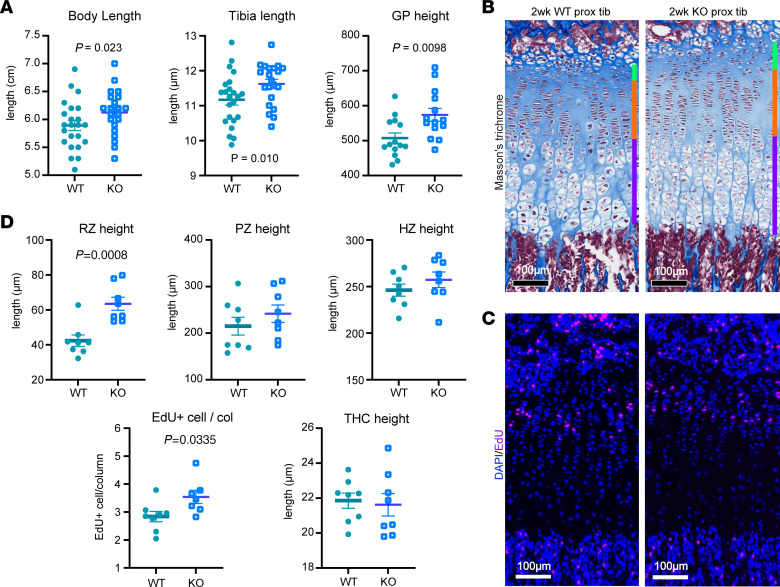
Quantitative histological analysis of 2-week-old WT and Spin4-KO male mice. (**A**) Body and tibia length (*n* = 18–22) and overall proximal tibia growth plate height (*n* = 14) were measured in 2-week-old WT and KO males. (**B**) Example of Masson’s trichrome–stained proximal tibial growth plate. Areas designated as resting, proliferative, and hypertrophic zones were labeled with green, orange, and purple lines, respectively, on the right side. Scale bar: 100 μm. (**C**) Example of EdU-labeled (in pink) DAPI counter-stained (in blue) proximal tibia growth plate at 2 weeks old. Scale bar: 100 μm. (**D**) Quantitative histological measurements of 2-week-old proximal tibial growth plate (*n* = 8). RZ, resting zone; PZ, proliferative zone; HZ, hypertrophic zone; THZ, terminal hypertrophic cell. *P* value represents Student’s *t* test.

Because loss of *SPIN4*/*Spin4* had a prominent effect on stature of the human subjects and on bone length in mice, we next focused on the growth plate, the cartilage responsible for bone elongation. We chose to study the mice at 2 weeks of age because, at this point, the secondary ossification center has formed, separating the growth plate from the articular cartilage, and skeletal growth is rapid at that age. We found that both body and tibial bone length were already significantly increased in the male KO mice compared with WT male littermates at this age (Figure 4A). In addition, 4 (liver, lung, heart, and brain) of the 6 organs studied showed increased weight (Supplemental Figure 3), suggesting that *Spin4* mutation was already having a measurable impact on organ growth at 2 weeks of age (Supplemental Figure 3). Again, we assessed a possible involvement of IGF-2 in the altered body growth, but neither serum IGF-2 levels nor mRNA levels of Igf2 or H19 (measured in liver and cultured growth plate chondrocytes) showed any significant difference between male KO mice and male WT littermates at 2 weeks of age (Supplemental Figures 4 and 5).

Histological analysis of the proximal tibial growth plate showed an overall increase in the growth plate height of the male KO mice (Figure 4, A and B), which prompted us to quantitatively analyze the different zones in the growth plate. The 3 zones of the growth plate — resting, proliferative, and hypertrophic — correspond to the stem-like progenitors, the actively dividing transit-amplifying cells, and the terminally differentiated collagen X–producing chondrocytes, respectively (16).

In the proliferative zone, we searched for effects that might contribute to the enhanced longitudinal bone growth of *Spin4-*KO mice. We saw no significant difference between KO and WT mice in the height of this zone, the number of proliferative cells per column, or the height of the individual cells (Figure 4D and Supplemental Figure 6). However, we did find an increase in the proliferation rate (assessed by EdU incorporation) of KO mice in this zone (Figure 4, C and D), and this increase likely contributes to the increase in longitudinal bone growth.

**Figure 5:**
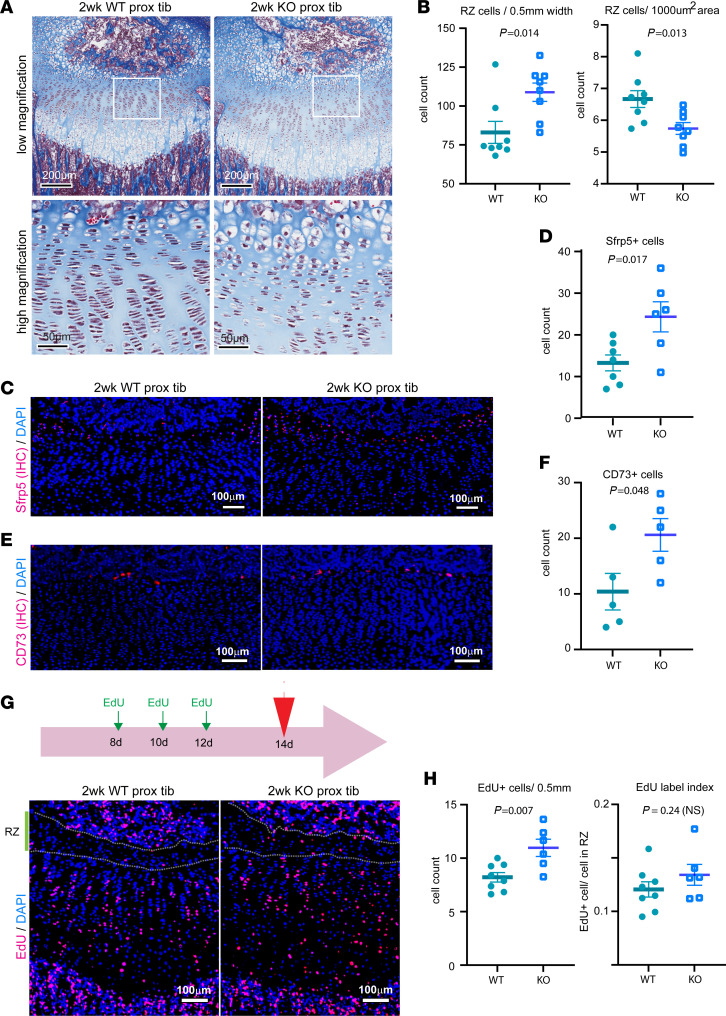
Resting zone is expanded in male mice with *Spin4* deletion. (**A**) Top panel: Masson trichrome–stained proximal tibia growth plate at 2 weeks old, highlighting the difference in resting zone. Scale bar: 200 μm. Bottom panel: high-magnification image of the inset from the top panel. Scale bar: 50 μm. (**B**) Number of resting zone chondrocytes was counted per 500 μm growth plate width (left) and normalized to the total area of resting zone measured (right). *n* = 8 (**C**–**F**) Immunofluorescence was used to compare the expression of progenitor cell markers Sfrp5 (*n* = 7) and CD73 (*n* = 5) in resting zone. (**G**) To study change in cell proliferation in the resting zone, we injected EdU at 8, 10, and 12 days of age, and growth plates were dissected at 14 days of age. Scale bar: 100 μm. (**H**) Overall EdU-labeled resting chondrocyte number was increased, but resting zone proliferative index (EdU count normalized to total cell number) was not statistically different between Spin4 KO and WT (*n* = 6-8). *P* value represents Student’s *t* test.

In the hypertrophic zone, we saw no significant difference between KO and WT mice in the height of the zone, the number of hypertrophic cells per column, or the height of the individual terminal hypertrophic cells (Figure 4D and Supplemental Figure 6). In the resting zone, the male KO mice showed a significant increase in zone height (Figure 4D). Because the resting zone contains the stem-like cells of the growth plate, we next asked whether the population of these progenitor cells was increased. We found that the number of resting zone chondrocytes and the number of cells expressing Sfrp5 and CD73, which serve as markers of resting zone progenitor cells (17, 18), were increased in KO mice (Figure 5, A and C–F).

We next hypothesized that this increase in resting zone cell number was due to increased proliferation of these cells. We therefore performed multiple EdU injections in 1-week-old mice and counted the number of EdU^+^ cells at 2 weeks of age (Figure 5G). We found an overall increase in EdU-labeled resting chondrocytes (Figure 5B). However, when the number of EdU^+^ cells was normalized to total cell number (yielding a proliferative index), the fraction of cells that incorporated EdU did not differ significantly between *Spin4*-KO and WT mice (Figure 5H). Our findings, therefore, do not support an increased tendency of progenitor cells to lose quiescence and increase proliferation at 1–2 weeks of age. It remains possible that the increased number of resting zone chondrocytes in KO mice may be due to an increased proliferation rate in earlier development or to reduced recruitment into the proliferative columns.

When resting zone chondrocyte number was normalized to area, the cell density was found to be decreased (Figure 5B), suggesting increased production or decreased resorption of cartilage extracellular matrix.

We also assessed *Spin4* expression in the WT growth plate and found a spatial gradient across the 3 zones (Supplemental Figure 7), with highest levels found in the resting zone (overall *P* < 0.05, pairwise comparison resting versus hypertrophic *P* = 0.033), which is consistent with the observed effects of *Spin4* KO in the resting and proliferative zones.

### Spin4 promotes Wnt signaling.

**Figure 6:**
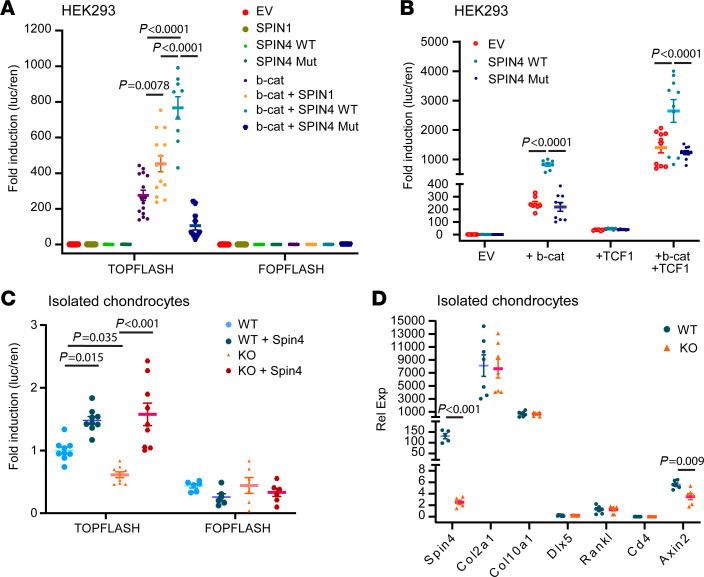
Spin4 promotes Wnt/β-catenin signaling. (**A**) In HEK293T cells, Spin1 and Spin4 WT (WT), but not frameshift variant (Mut), promotes Wnt signaling when cotransfected with β-catenin (1-way ANOVA; *n* = 10–15). Signals were represented in a luciferase activity ratio of firefly luciferase (from TOPFLASH or FOPFLASH) over renilla luciferase (for normalization of transfection efficiency). (**B**) SPIN4 WT, but not SPIN4 Mut, increased TOPFLASH signal when cotransfected with TCF1 and β-catenin. The induction was not observed, however, when cotransfected with TCF1 alone, suggesting the dependence on β-catenin expression (1-way ANOVA, *n* = 8–10). (**C**) In monolayer chondrocytes isolated from 1-week-old WT mice or Spin4 long deletion (KO), luciferase assay of TOPFLASH showed decreased baseline Wnt signaling activity in KO cells. This difference was abolished when mouse Spin4 was overexpressed in both cells, suggesting that the difference was due to a lack of endogenous Spin4 expression (1-way ANOVA, *n* = 9). (**D**) KO chondrocytes showed decreased expression of *Spin4* and *Axin2* but similar expression levels of *Col2a1* and *Col10a1* compared with WT chondrocytes. Expression of *Dlx5*, *Rankl*, and *Cd4* were low, suggesting minimal contamination with osteoblast or bone marrow cells (Student’s *t* test, *n* = 5–7).

We sought to explore the molecular mechanisms that contribute to the increased number of chondrocytes in the resting zone of *Spin4*-KO mice. Previous studies have shown that resting zone chondrocytes are maintained in a Wnt-inhibitory environment and that aberrant canonical Wnt/β-catenin signal activation is associated with a narrowed and dysfunctional resting zone (19). Moreover, another member in the spindlin family, SPIN1, was previously shown to promote Wnt/β-catenin signaling (11, 20, 21). Based on these previous studies, we hypothesized that SPIN4 similarly promotes Wnt/β-catenin signaling and that consequently *Spin4* KO decreases Wnt signaling, thereby promoting maintenance of the resting zone. We first tested whether *SPIN4* can promote Wnt signaling in HEK293 cells using a TOPFLASH luciferase reporter (22). Contrary to previous studies (20), we did not see any significant induction of TOPFLASH signal upon *SPIN1* or *SPIN4* expression (Figure 6A). However, when we cotransfected *SPIN1* or *SPIN4* with a β-catenin–expressing plasmid, we saw significant induction of TOPFLASH signal compared with β-catenin alone (Figure 6A). The luciferase induction was particularly robust with WT *SPIN4* (~800-fold versus ~250-fold for β-catenin, *P* < 0.0001). This effect was not seen with the frameshift *SPIN4* variant (Figure 6A). We next explored the involvement of TCF1, a transcription factor that recognizes TCF binding sites (present in the TOPFLASH plasmid) and also binds β-catenin to promote transcription of Wnt-responsive target genes. When cells were transfected with TCF1 instead of β-catenin, *SPIN4* did not increase TOPFLASH signal. However, when cells were transfected with TCF1 and β-catenin together, WT *SPIN4* was able to induce TOPFLASH signal (Figure 6B). Thus, the induction by SPIN4 appeared primarily dependent on β-catenin, rather than TCF1. Taken together, these findings suggest that WT SPIN4 promotes canonical Wnt signaling and that the frameshift variant abolishes this effect.

### Decreased Wnt signaling in chondrocytes from Spin4-KO mice.

Next, we investigated whether Wnt signaling is altered in chondrocytes from *Spin4*-KO mice. We, therefore, isolated growth plate chondrocytes from 1-week-old WT or *Spin4-*KO (long-deletion) mice and measured Wnt activity using TOPFLASH. Interestingly, we found that *Spin4*-KO chondrocytes had reduced baseline Wnt activity compared with WT chondrocytes (Figure 6C). This difference was abolished when mouse *Spin4* was overexpressed in WT and KO cells (Figure 6C), suggesting that the previously observed difference in Wnt signaling between WT and KO cells was due to a lack of endogenous *Spin4*. WT and KO chondrocytes showed similar expression levels of collagen 2 (*Col2a1*) and collagen X (*Col10a1*) and very low levels of *Dlx5*, *Rankl*, and *Cd4*, indicating minimal contamination with osteoblast or bone marrow cells (Figure 6D). Interestingly, *Axin2* mRNA expression, a readout for Wnt signaling activity, was also decreased in chondrocytes from the *Spin4-*KO mice (Figure 6D), and it further supports a reduced baseline Wnt activity compared with WT chondrocytes. These findings indicate that the difference in baseline Wnt activity is not likely caused by a difference in the preparation of isolated chondrocytes. Overall, the findings in cultured growth plate chondrocytes are consistent with the hypothesis that loss of *Spin4* decreases Wnt signaling in growth plate chondrocytes and that this Wnt-inhibitory environment is in favor of maintaining the resting zone.

## Discussion

In children, impaired body growth is a common finding and can be caused by a wide variety of hormonal, nutritional, and genetic disorders. In contrast, overgrowth disorders are far less common, and there are far fewer known causes. We studied a boy with generalized overgrowth including macrocephaly, hepatosplenomegaly, and extreme tall stature who carried a hemizygous frameshift variant in *SPIN4*. We found multiple lines of evidence indicating that this variant caused the overgrowth phenotype, including (a) the variant segregated with tall stature in the family, in that it was present in the mother and maternal grandmother — both of whom were tall for their genetic background — but not in the remaining family members who reached their expected height; (b) the variant is a 2 bp deletion that leads to a frameshift and early protein truncation; (c) neither this variant nor any other protein-truncating variants have been reported in gnomAD, a large population database; (d) the identified frameshift variant abolished SPIN4 function, in terms of its ability to inhibit proliferation in cultured cells, bind specifically modified histones, and stimulate Wnt signaling; and (e) in mice, truncating variants in *Spin4* caused generalized overgrowth, recapitulating the human phenotype.

The phenotype of the proband provides valuable clues about the role of SPIN4 in regulating childhood growth. The observed tall stature, macrocephaly, and hepatosplenomegaly suggest that SPIN4, an epigenetic reader, normally serves to inhibit growth of multiple tissues, including growth plate cartilage, brain, liver, and spleen. The unusually large body size at birth indicates that SPIN4 normally regulates fetal as well as postnatal human growth. Interestingly, similar generalized overgrowth phenotypes are caused by inactivating variants in other genes that participate in epigenetic regulatory pathways mostly as epigenetic writers, including *EZH2* (Weaver syndrome) (5), *EED* (Cohen-Gibson syndrome) (23), *SUZ12* (Imagawa-Matsumoto syndrome) (24), *NSD1* (Sotos syndrome) (6), and *DNMT3A* (Tatton-Brown-Rahman syndrome) (7). *EZH2*, *EED*, and *SUZ12* are components of the polycomb repressive complex 2 (PRC2), and this likely explains why genetic changes in these genes cause similar overgrowth syndromes (25).

Patients with these overgrowth syndromes involving epigenetic mechanisms often present with developmental delay or mild to moderate intellectual disability. However, the proband lacking functional *SPIN4* in our study did not have developmental delay or cognitive impairment. Moreover, patients with other overgrowth syndromes involving epigenetic mechanisms often have advanced bone age, which reduces the total period for linear growth during childhood, resulting in a normal height rather than tall stature as an adult. Interestingly, our proband did not show a significantly advanced bone age, giving him an adult height prediction of +3.7 SDS (+3.3 gain to his midparental height). The hemizygous male proband in our study showed a more severe phenotype than either his heterozygous mother or maternal grandmother, a pattern common for X-linked conditions. Accumulating additional subjects with *SPIN4* variants in future studies will clarify the phenotypic spectrum in males and females.

To explore the role of SPIN4 in mammalian growth further, we introduced truncating mutations in *Spin4* in mice. Loss of *Spin4* recapitulated the human disorder, increasing growth of multiple organs, including the kidney, heart, lung, brain, spleen, and skeleton. Thus, the role of SPIN4/Spin4 has been well conserved during mammalian evolution.

To understand better the cellular basis for the skeletal overgrowth, we focused on the growth plate, a cartilaginous structure responsible for bone elongation. We found that the transit-amplifying cells, which are located in the proliferative zone, showed an increased proliferation rate, whereas the terminally differentiated cells of the hypertrophic zone were of normal size, suggesting that the somatic overgrowth, at least in this tissue, is due to hyperplasia rather than hypertrophy. Interestingly, the number of stem-like cells of the resting zone were increased, suggesting that Spin4 negatively regulates maintenance of this stem-like pool. We also manipulated expression of SPIN4 in isolated cell culture, finding that knockdown increased proliferation while overexpression decreased proliferation, consistent with the hypothesis that the proliferative effect of Spin4 is cell autonomous and not dependent on endocrine or paracrine signals, but establishing that a cell-autonomous effect on cell proliferation is responsible for the somatic overgrowth in vivo will require additional studies.

We next explored the molecular mechanisms by which SPIN4 acts. Previous studies indicate that SPIN1, another member in the spindlin family, binds specifically modified histones (10). There is also evidence that other spindlin family members, including *SPIN2B*, *SPIN3*, and *SPIN4*, share some histone binding capacity (14). Using histone peptide arrays, we confirmed that SPIN4 binds modified histones, such as H3K4me3 and H4K20me3. Fluorescence microscopy studies indicated nuclear localization, and subcellular fractionation further suggested chromatin binding by SPIN4, supporting the hypothesis that the observed histone binding occurs in cells. These data suggest that SPIN4 serves as a histone reader, recognizing epigenetic chromatin marks to mediate downstream cellular effects. In contrast, genes previously implicated in overgrowth disorders, such as *EZH2*, *NSD1*, and *DNMT3A*, serve primarily as epigenetic writers; therefore, to our knowledge, *SPIN4* is the first gene implicated in an overgrowth disorder that appears to act primarily as an epigenetic reader. It is not clear why these various genetic abnormalities, which affect such disparate epigenetic pathways, have such a similar clinical phenotype. Further studies will be required to reach an understanding of the coordination of epigenetic writers, readers, and erasers in the regulation of growth.

Because SPIN1 promotes canonical Wnt signaling (12, 20), we hypothesized that SPIN4 might also serve as an activator of this pathway. In the canonical pathway, WNT signaling leads to stabilization of β-catenin, which translocates into the nucleus and forms nuclear complexes with TCF family members. SPIN1 binds TCF-4 to activate transcription of WNT-responsive target genes (12). We found that, in HEK293 cells overexpressing β-catenin, SPIN4 similarly promotes Wnt signaling. We also found evidence that growth plate chondrocytes lacking *Spin4* show reduced basal Wnt signaling. Previous studies suggest that the stem-like cells of the resting zone are maintained in a WNT-inhibitory environment (19) and, therefore, the observed decrease in chondrocyte Wnt signaling may explain the increased number of resting zone chondrocytes in *Spin4*-KO mice. WNT signaling plays a critical role in stem cell regulation, not only in growth plate cartilage, but in multiple tissues (26), raising the possibility that the observed overgrowth of other *SPIN4*-deficient tissue may be mediated, in part, by altered stem cell function.

Spin4, like other members of the Spin family, contains 3 tandem Tudor-like domains. These conserved motifs bind methylated lysine and arginine residues in target proteins and, thus, play important roles in reading chromatin marks. Prior studies showed that Tudor-like domains 1 and 2 in Spin1, which correspond to domain 1 and 2 in Spin4, are important for recognition of specific histone modifications and that these domains are likely involved in activating transcription of Wnt/β-catenin target genes (10). Our current findings further suggest that Tudor domain 3 may also be important in Spin4 activity because, in our mouse model with the short *Spin4* deletion, the ablation of Tudor-like domain 3 alone was sufficient to promote growth in vivo and to impair histone binding in vitro.

There has been a paucity of studies showing how genetic changes in epigenetic regulatory proteins cause skeletal overgrowth. In humans, heterozygous loss-of-function variants in *NSD1* increase skeletal growth in Sotos syndrome, whereas, in mice, homozygous loss of *Nsd1* impairs skeletal growth, in part by regulating *Sox9* expression in chondrocytes (27). Similarly, in humans, heterozygous partial loss-of-function variants in *EZH2* increase skeletal growth in Weaver syndrome (28), whereas, in mice, homozygous loss of *Ezh1* and *Ezh2* impairs growth plate function by inhibiting proliferation and hypertrophy of growth plate chondrocytes (29). There is also evidence that *EZH2* may work through the Wnt/β-catenin pathway (30). Whether the loss of *EZH2* might decrease Wnt/β-catenin signaling — thus, supporting maintenance of the resting zone causing skeletal overgrowth — will require future studies. *DNMT3A* has been shown to inhibit Wnt/β-catenin signaling in cardiac progenitor cells (31). Therefore, the mechanism of skeletal overgrowth caused by *DNMT3A* variants may differ from that of S*PIN4*.

Taken together, our findings indicate that S*PIN4* negatively regulates body growth in humans and mice and that loss-of-function variants in *SPIN4* cause a human generalized overgrowth syndrome. Our findings further suggest that SPIN4 normally acts to recognize specifically modified histones, to promote WNT signaling, and to restrain cell proliferation. Although many of our mechanistic studies focused on the growth plate, similar overgrowth was seen in other organs lacking Spin4, suggesting that Spin4 normally regulates growth in a broad range of tissues. To our knowledge, *SPIN4* is the first gene implicated in an overgrowth disorder that appears to act primarily as an epigenetic reader.

## Methods

### Patient report and genetic analysis.

Whole bloods from the proband and his family members (maternal grandmother, mother, father, and half-sister) were collected for SNP array and exome sequencing. The method of SNP array, exome sequencing, and data analysis are described in our previous studies (32).

### Animal procedures and generation of Spin4-KO mice.

Mice carrying a truncating mutation of *Spin4* were created by CRISPR-mediated genome editing directly in fertilized eggs of C57BL/J (The Jackson Laboratory) as described (33). Guide RNAs (gRNAs) targeting *Spin4* with minimized predicted off-target hits were initially selected using the ranking tool CRISPR Design (http://crispr.mit.edu) and CRISPRScan.org, synthesized with the MegaScript T7 kit (Ambion), and efficacy was confirmed with Surveyor assay. Sequences of the 2 gRNAs used were 5′-GCCTCCTACTGGGGTGGA-3′ and 5′-GGTGCTCGACAGCCTTGT-3′. The 2 gRNAs were loaded onto SpCas9 protein by incubating at 37°C for 15 minutes and microinjected into fertilized eggs isolated from C57BL6/J donor females (The Jackson Laboratory). The injection dosage of the gRNA was titrated to the minimal effective concentration, 1 ng/μL in this case. Genomic DNA was obtained from the tail snips of F0 mice. A PCR amplicon spanning the intended deletion region was generated using primers forward, 5′-TCAGTTCCCAGTCAGTTAGC-3′; and reverse 5′-ACACGTTTCGCAAAGAGGTCTAT-3′. WT mice produced an amplicon of 935 bp, while mice carrying a truncating mutation of *Spin4* produced a band of reduced molecular size. We chose to characterize 2 strains of mice (Supplemental Figure 8), one with a truncating mutation downstream of Tudor domain 2 (producing an amplicon of 824 bp) and another with a truncating mutation essentially removing all 3 Tudor domains (producing an amplicon of 421 bp).

### Plasmid preparation.

Plasmids expressing GFP-tagged, Fc-tagged, or FLAG-tagged SPIN1, WT, or mutant SPIN4 were constructed by PCR amplification of gene fragment using Q5 High-Fidelity DNA Polymerase (New England Biolabs), followed by Gibson assembly using HiFi assembly master mix (New England Biolabs). The plasmids used for the backbone included pEGFPN1 (Clontech), pSecTag (Invitrogen), and pcDNA3.1 (Invitrogen). Plasmid expressing H2B-GFP was a gift from Geoff Wahl (The Salk Institute for Biological Studies, La Jolla, California, USA; Addgene, plasmid no. 11680) (34). TOPFLASH and FOPFLASH plasmids were a gift from Randall Moon (University of Washington, Seattle, Washington, USA; Addgene, plasmid nos. 12456 and 12457) (22). pcDNA3-HA-TCF1 was a gift from Kai Ge (NIDDK; Addgene, plasmid no. 40620) (35).

### Cell culture.

HEK293T cells were obtained from ATCC (CRL-3216, ATCC) and maintained in DMEM (Thermo Fisher Scientific) supplemented with 10% FBS (v/v) (Thermo Fisher Scientific) and 1% penicillin-streptomycin (PS) (v/v) (Thermo Fisher Scientific) at 37°C in a humidified atmosphere of 5% CO_2_. Expi293F cells (A14528, Thermo Fisher Scientific) were cultured in suspension in Expi293 Expression Medium (Thermo Fisher Scientific) shaking at 100–130 rpm in a Multitron Humidified Incubator Shaker (INFORS HT) maintained at 37°C and 8% CO_2_.

### Chondrocyte isolation and culture.

Growth plates from proximal tibias and distal femurs were dissected aseptically from 1-week-old mice and digested in 0.3% collagenase type I (Sigma-Aldrich) in DMEM/F12 medium. For monolayer culture, the liberated cells were resuspended and plated at a density of 1 × 10^5^ chondrocytes per well in 12-well-plates, in DMEM/F12 medium (Invitrogen) supplemented with 10% FBS, 1% penicillin (100 U/mL)/streptomycin (100 μg/mL), and 50 μg/mL ascorbic acid in a humidified incubator at 37°C, 5% CO_2_.

### Cell transfection.

HEK293T cells were transfected with Lipofectamine 2000 (Thermo Fisher Scientific) according to the manufacturer’s protocol as previously described (36). Expi293 cells were transfected with Turbo293 Transfection Reagent (SPEED Biosystems) according to the manufacturer’s protocol. Monolayer chondrocytes were transfected as previous described (29). Briefly, 1 day after 1 × 10^5^ chondrocytes were plated per well in 12-well plates, hyaluronidase (5 U/mL, Sigma-Aldrich) was added to the cultured cells for 4–6 hours. One hour prior to transfection, cells were washed once in PBS and changed to DMEM/F12 medium with 10% FBS without antibiotics. Chondrocytes were then transfected with Lipofectamine 2000 (Thermo Fisher Scientific) following the manufacturer’s protocol. Cells were changed back to regular culture medium the next day after transfection and were cultured for an additional 24 hours for downstream assay.

### Subcellular fractionation.

Cytoplasmic, nuclear, and chromatin-bound proteins from HEK293T cells were prepared using Subcellular Protein Fractionation Kit (Thermo Fisher Scientific) according to the manufacturer’s standard protocol. In general, 1.5 × 10^6^ HEK293T cells were plated on 10 cm^2^ culture dishes and transfected the next day. Then, at 48 hours after transfection, cells were trypsinized, harvested, and counted. Five million to 10 million cells were typically used for subcellular fractionation, and protein concentrations were assessed by BCA reagent (Thermo Fisher Scientific) using s standard curve.

### Western blot.

Protein was isolated from HEK293T using RIPA buffer supplemented with a proteinase inhibitor cocktail (Sigma-Aldrich) and PhosSTOP (Sigma-Aldrich). Cell lysates were incubated on ice for 30 minutes, followed by a 10-minute centrifugation at 21,000*g* at 4°C to remove cell debris. Western blotting was performed as previously described (37) using anti-FLAG antibody (MilliporeSigma, F3165), anti-GFP (Enzo Biochem, ALX-210-199-R100), or anti-histone H3 (Abcam, ab18521).

### Proliferation assessment by ^3^H-thymidine uptake.

HEK293T cells were cultured and transfected with plasmids or siRNA. After 24 hours, the cells were incubated with fresh culture medium containing 1 μCi of ^3^H-thymidine (72 μCi/mL, MP Biomedicals) for 24 hours. After incubation, cells were washed with PBS and collected by trypsinization (0.25%, 5 minutes) to measure radioactivity by liquid scintillation counting.

### Purification of Fc-tagged SPIN4 proteins.

WT SPIN1, WT SPIN4, or mutant SPIN4 coding sequence were cloned into pSecTag plasmid containing a secretary peptide sequence so that the expressed proteins are secreted into the culture media. An antibody Fc region was inserted at the carboxyl terminus, connected with a G4S linker (38) to allow protein purification using protein A resin (GenScript) as previously described (39). Briefly, protein A resin slurry (1–5 mL) was packed into a glass Econo-column (Bio-Rad) and equilibrated with 50 mL of binding/washing buffer (0.15M NaCl, 20 mM Na_2_HPO_4_ [pH 8.0]). Four to 6 days after transfection, Expi293 cells were spun down, and the culture media containing the expressed protein was loaded onto the column. After unbound proteins were washed away with binding/washing buffer, SPIN1 or SPIN4-Fc fusion proteins were eluted with 30 mL of elution buffer (100 mM acetic acid [pH 3.0]), neutralized by 1/10 volume of neutralization buffer (1M Tris-HCl [pH 9.0]), concentrated, and buffer-exchanged to PBS using Amicon Ultra-15 filter unit (MilliporeSigma). The purity of the antibodies was checked by SDS-PAGE, and the concentration was determined by Nanodrop (Thermo Fisher Scientific).

### Histone array.

MODified Histone Peptide Array (Active Motif) was used to assess the binding of SPIN1 and SPIN4 to modified histones. Array slides were blocked overnight with 5% milk and 0.1% TBS-T at 4°C. After blocking, slides were washed 3 times for 15 minutes with 0.1% TBS-T. Purified SPIN4-Fc fusion proteins were then diluted in buffer contain 150 mM NaCl, 20 mM Tris-HCl (pH8.0), and 0.1% NP-40. We used 5 nmol of SPIN4-Fc protein (or 270 μg protein with a molecular weight of 54 kDa) in 10 mL of buffer. Diluted proteins were added to the histone array and incubated overnight at 4°C with gentle rocking. After incubation, the array slides were washed 3 times with 0.1% TBS-T, followed by incubation with HRP-conjugated anti-Fc antibody (MilliporeSigma, AP113P) in 5% milk and 0.1% TBS-T at room temperature (r.t.) for 2 hours. The array slides were again washed 3 times with 0.1% TBS-T, and signals were developed with ECL prime reagent (Cytivia Life Sciences) and imaged with Amersham Imager 680 (Cytivia Life Sciences). Images were analyzed using the Array Analyze Software provided by Active Motif.

### Growth plate histology.

Masson trichrome–stained proximal tibia epiphyseal sections were used for histological evaluation of the growth plates, which were visualized with a ScanScope CS digital scanner (Aperio Technologies Inc.) under bright-field microscopy. All histological measurements were performed in the central two-thirds of the growth plate sections as previously described (40). At 2 weeks of age, the beginning of the resting zone was defined as the lower margin of the secondary ossification center. The number of cells in the resting zone was counted per 500 μm growth plate width. For each growth plate section, we performed at least 5 measurements of resting zone height, resting zone cell count, proliferative zone height, hypertrophic zone height, and terminal hypertrophic cell height. For the evaluation of terminal hypertrophic cell height, the height of the lacunae — which reflects the actual hypertrophic chondrocyte height before cells condense during tissue fixation and processing — was measured. For each animal, averages were taken from a minimum of 20 measurements from 4 growth plate sections.

### Immunostaining and fluorescence microscopy.

We followed protocols for immunostaining the growth plate from prior studies (18, 41) with some modifications. After animals were sacrificed, tibial and femoral epiphyses were excised and fixed overnight in 10% formalin at 4°C and decalcified in 10% (w/v) EDTA (pH 7.4) with 5% formalin. For paraffin-embedded sections, bones were decalcified for 2 weeks or longer and sent to Histoserv (Germantown, Maryland, USA) for sectioning. For frozen sections, bones were decalcified for 3 days, cryoprotected by immersing in phosphate-buffered saline (PBS) with 30% (w/v) sucrose overnight, and then embedded in OCT and frozen in Tissue-Tek Cryo 3 Flex Cryostat for sectioning. To detect CD73-expressing cells, 25 μm frozen sections were baked at 65°C for 1 hour and postfixed in 1% formaldehyde for 10 minutes. Antigen retrieval was performed by treating with 0.1%Trypsin for 30 minutes at r.t. Slides were blocked in 3% horse serum were then incubated with Alexa Fluor 647–conjugated mouse anti-CD73 (BioLegend, 127208, dilution 1:30) overnight at 4°C. After washing, slides were incubated in a DAPI solution (300 nM, in 0.1% TBS-T) for 5 minutes at r.t. to visualize the cell nuclei and were mounted with ProLong Diamond AntiFade Mountant (Thermo Fisher Scientific). To detect Sfrp5-expressing cells, 10 μm paraffin-embedded sections were baked at 65°C for 1 hour, deparaffinized with xylene, and rehydrated with ethanol steps. Antigen retrieval was performed by heating the slides in citric acid–based antigen-unmasking solution (Vector Laboratories) to 99°C for 10 minutes, followed by a 20-minute cooldown. Slides were then blocked with 10% goat serum and were then incubated with rabbit anti-Sfrp5 (Abcam, ab230425, dilution 1:20) as primary antibody and Alexa Fluor 647–conjugated goat anti–rabbit IgG (Thermo Fisher Scientific, A32733, dilution 1:2,000) as secondary antibody. Slides were scanned with a Keyence BZ-X700 fluorescence microscope (Keyence Corp.) at 10× magnification. For quantification, the number of Alexa Fluor 647^+^ cells were counted and normalized to the width of the metaphyseal bone (per 500 μm).

### EdU staining and analysis.

The proliferation rate was determined by EdU (Thermo Fisher Scientific) staining. For labeling the proliferative zone, EdU was injected (100 mg/kg body mass i.p.) in 2-week-old mice. 2 hours later, the mice were killed, and the growth plates were dissected, fixed, and decalcified. For labeling the resting zone, EdU was injected at 8, 10, and 12 days old, and mice were sacrificed at 14 days old. Samples were cryopreserved in 30% sucrose, embedded in OCT compound, and frozen at –20°C. Sections (10 μm) were obtained and placed on Superfrost Plus slides. EdU labeling was detected by IHC using Click-iT Alexa Fluor 488 EdU Cell Proliferation Kit (Thermo Fisher Scientific) and counterstained with DAPI. Slides were examined using a Keyence BZ-X700 fluorescence microscope (Keyence Corp.) at 20× magnification using a GFP filter (ex470/em525) for EdU 488 and a DAPI filter (ex360/em460). EdU^+^ cells were counted in the center two-thirds of the growth plate proliferative zone, and the counted number was divided by the total number of proliferative columns in the counted area. For resting zone labeling, EdU^+^ cells were counted in the center two-thirds of the growth plate resting zone and normalized to the width of the metaphyseal bone (per 500 μm) and to the total cell count (resting zone proliferative index).

### Laser capture microdissection (LCM).

One-week-old WT C57BL/6 male mice were sacrificed and proximal tibias were excised and embedded in OCT compound, frozen on dry ice, and stored at –80°C. LCM of growth plate cartilage was performed as previously described (42). RNA extraction was performed using the RNeasy Micro Kit (QIAGEN). For quality control purposes, we used real-time PCR to assess expression levels of Pthlh, Gdf10, and Col10a1, which serve as zonal markers of the resting, proliferative, and hypertrophic zone, respectively, to confirm lack of cross contamination between different zones of the growth plate during dissection.

### RNA extraction and purification.

RNA was extracted from monolayer chondrocytes or 2-week-old mouse liver tissue using an RNeasy Mini Kit (QIAGEN). All RNA samples had a 260/280 nm ratio between 1.8, and 2.1. RNA integrity was determined using an Agilent 2100 Bioanalyzer (Agilent Technologies). All RNA samples have good RNA quality, typically with RIN number of 7–8.

### qPCR.

Real-time PCR was used to study gene expression in chondrocytes. Total RNA (50–100 ng) was reverse transcribed using SuperScript IV Reverse Transcriptase (Invitrogen). qPCR was performed as previously described (43) using commercially available FAM- or VIC-labeled TaqMan assays (Applied Biosystems). For Spin4, SYBR Green–based reaction was used with the following primers: forward, 5′-CCCTGCTGGACGACTACAAA-3′; reverse, 5′-TGTTCAGCTGCAGGGAAGTAG-3′. Reactions were performed in triplicate on cDNA derived from each animal using the ABI QuantStudio 6 Flex System instrument (Applied Biosystems). The relative quantity of each mRNA was calculated using the formula, Relative Expression = 2^−ΔCt^ × 10^6^, where Ct represents the threshold cycle and ΔCt = (Ct of gene of interest) − (Ct of 18S rRNA). Values were multiplied by 1 × 10^6^ for convenience of comparison.

### Luciferase assay.

HEK293T or chondrocytes were cultured and transfected as described above. For luciferase assay to assess Wnt signaling activity (22), cells were cotransfected with 10 ng of pRL Renilla luciferase reporter (for normalization of transfection efficiency), 200 ng of TOPFLASH TCF reporter plasmid (or FOPFLASH, the mutated version of TOPFLASH), and different combinations of expression plasmids, such as pcDNA-SPIN4-FLAG, pcDNA-TCF1-HA, pcDNA-β-catenin, or pcDNA alone at 200 ng each in 24-well plates. Cells were lysed 48 hours after transfection, and luciferase expression was assessed using the Dual-Luciferase Reporter Assay System (Promega) and a spectrophotometer (36).

### Serum IGF2 measurement.

To measure IGF2 levels in mouse serum, we anesthetized mice at 2 weeks of age by injection of ketamine (100 mg/kg) and xylazine (5 mg/kg). Blood was collected using cardiac puncture and allowed to sit at r.t. for 2 hours; it was then centrifuged at 2,000*g* for 20 minutes at room temperature. IGF-2 levels in mouse serum was then measured by mouse/rat/porcine/canine IGF-II/IGF2 Quantikine ELISA Kit (R&D Systems) following the manufacturer’s protocol.

### Statistics.

The exact sample size (*n*) for each experimental group/condition, wherever applicable, was provided in the figure legends. All distinct data points shown in the paper were taken from distinct samples (biological replicates) rather than from the same sample measured repeatedly (technical repeats). Unless otherwise stated, statistical comparisons were done with 2-tailed Student’s *t* test (when comparing 2 groups) or 1-way ANOVA (when comparing multiple groups) with correction of multiple comparisons (Bonferroni test, whenever applicable). A *P* value (after correction for multiple comparisons, if applicable) of less than 0.05 was considered significant.

### Study approval.

The study on human subjects was approved by the NICHD IRB. All adult subjects and parents of minors provided written informed consent, and children provided written assent. All animals were used in accordance with *Guide for the Care and Use of Laboratory Animals* (National Academies Press, 2011) and approached by NICHD IACUC.

## Author contribution

YHJ and JB conceived the project. YHJ contributed to patient care and exome sequencing data analysis. JCL and JB contributed to experimental design. JCL, JW, and EZ performed experiments and analyzed data. LD created the mouse model. KMB contributed to mouse husbandry. JCL, YHJ, and JB wrote the manuscript.

## Supplementary Material

Supplemental data

## Version 1 03/16/2023

In-Press Preview

## Version 2 05/08/2023

Electronic publication
